# The association of Big-Five personality with co-rumination and its trade-off effect in Chinese adolescents: a cross-sectional study

**DOI:** 10.3389/fpsyg.2025.1603507

**Published:** 2025-09-11

**Authors:** Zilong Luo, Pinchao Luo

**Affiliations:** ^1^School of Psychology, South China Normal University, Guangzhou, China; ^2^Center for Studies of Psychological Application, South China Normal University, Guangzhou, China; ^3^Key Laboratory of Brain, Cognition and Education Sciences, South China Normal University, Guangzhou, China; ^4^Guangdong Key Laboratory of Mental Health and Cognitive Science, South China Normal University, Guangzhou, China

**Keywords:** co-rumination, anxiety, depression, Chinese adolescents, Big Five, friendship quality

## Abstract

**Introduction:**

Co-rumination, the excessive discussion of negative events with peers, exhibits a trade-off effect: it enhances friendship quality while simultaneously increasing the risk of internalizing problems in adolescents. Previous research has demonstrated group differences in this trade-off effect. Building on prior findings, this study explores the relationship between the Big Five personality traits and co-rumination among Chinese adolescents through three analytical components, aiming to identify at-risk groups for co-rumination using personality traits.

**Methods:**

This cross-sectional study collected 765 valid self-reported responses. Measurement invariance of the Chinese version of the co-rumination questionnaire was examined, and latent variable structural equation models were constructed for each of the three research objectives to investigate the relationship between the Big Five traits and co-rumination.

**Results:**

First, measurement invariance of the co-rumination questionnaire was assessed, revealing scalar invariance across gender and partial scalar invariance across age groups. Subsequent analyses examined the relationship between the Big Five traits and co-rumination. The first part showed that extraversion, neuroticism, and openness positively predicted co-rumination, while neuroticism explained gender differences in co-rumination. The second part confirmed the moderated trade-off effect of co-rumination; after controlling for the Big Five traits, the risk pathway of co-rumination became more robust, with gender differences observed in this pathway. The third part revealed distinct moderating effects of neuroticism, agreeableness, extraversion, and conscientiousness on the risk pathway.

**Discussion:**

Given the measurement invariance results, the Chinese co-rumination questionnaire should be used more cautiously in studies involving multiple age groups. Structural equation modeling indicated that neuroticism serves as a strong indicator for identifying at-risk groups, as individuals with high neuroticism are more susceptible to risks associated with co-rumination. High conscientiousness and agreeableness were found to buffer against co-rumination-related depression and anxiety, respectively. The roles of openness and extraversion in relation to co-rumination appear more complex. Further research is needed to validate these findings.

## 1 Introduction

Co-rumination is viewed as a form of communication involving excessive discussion of negative topics in dyadic interactions between two individuals, considered a type of self-disclosure with ruminative property (Rose, 2002). Co-rumination among adolescents presents a trade-off effect, as it may have both benefits and risks. Research shows that co-rumination can enhance friendship quality and intimacy. Good peer friendship quality may serve as a protective factor against internalizing problems (La Greca and Harrison, 2005). However, co-rumination may also exacerbate internalizing symptoms, such as depression and anxiety, by intensifying post-event brooding (Bastin et al., 2021). The extent of co-rumination and its accompanying trade-off effects vary based on adolescents’ gender and social developmental stages, though prior findings remain inconsistent regarding certain group differences in co-rumination. Furthermore, recent studies have identified group disparities in the benefits and risks of adolescent co-rumination, with some high-risk individuals more prone to developing internalizing symptoms rather than improved friendship quality (DiGiovanni et al., 2021; Tilton-Weaver and Rose, 2023). While research has attempted to explore factors identifying these at-risk groups, personality factors have not been explicitly considered. To address these gaps, the present study employs a cross-sectional design, utilizing a rarely examined sample of Chinese adolescents—a population with typical collectivist cultural traits—in co-rumination research. Through more precise latent variable analysis, this study is the first to conduct cross-group measurement invariance validation for the co-rumination questionnaire while re-examining established or conflicting prior findings. Additionally, it comprehensively explores, for the first time, the relationship between the Big Five personality traits and co-rumination and its associated outcomes, aiming to identify personality indicators that may help detect high-risk co-rumination groups.

### 1.1 Co-rumination

Co-rumination research primarily focuses on children and adolescent groups. As individuals develop, especially during adolescence, peers become a crucial source of social support for teenagers. Their social interaction patterns with friends become more complex and frequent, with different interaction styles observed across gender (Rose and Rudolph, 2006). These age and gender differences in social interaction patterns are also reflected in co-rumination, which is a dyadic interactive structure. In terms of gender, girls tend to engage in more co-rumination than boys. Regarding age, from childhood to adolescence, as individuals grow older, they engage in more discussions about personal troubles with friends, leading to increased co-rumination (Rose, 2002). This study will examine the measurement invariance of the co-rumination questionnaire and employ more precise latent mean comparisons to validate these cross-group differences.

### 1.2 Co-rumination and internalizing symptoms

Co-rumination involves trade-offs, bringing both risks and benefits. Previous research has shown that co-rumination leads to internalizing problems in adolescents, such as depression and anxiety. Schwartz-Mette and Rose (2012) found that co-rumination facilitates the contagion of depressive and anxiety symptoms among peers. Dirghangi et al. (2015) employing a monozygotic twin design to control for genetic and shared environmental factors, demonstrated that individual differences in co-rumination significantly predicted variations in anxiety. Researchers have identified the internal mechanisms linking co-rumination to internalizing symptoms. Specifically, adolescents engaging in co-rumination with peers experience heightened post-event brooding, which amplifies negative emotions and ultimately contributes to internalizing problems (Bastin et al., 2021).

While there is consensus that co-rumination—a social interaction behavior—varies across developmental stages and gender, its association with internalizing problems remains contentious. For example, Rose et al. (2007) reported that co-rumination predicted depression and anxiety exclusively in female adolescents. In contrast, Schwartz-Mette and Rose (2012) observed no gender or age differences in the contagion effects of co-rumination on depressive symptoms, though anxiety contagion was limited to females and adolescents (compared to children). However, some studies have failed to identify moderating effects of gender or age on the co-rumination–internalizing symptom relationship (Bastin et al., 2021). Given these contradictions, the current study utilizes a Chinese adolescent sample to examine the associations between co-rumination and depression/anxiety, and the potential moderating roles of gender and age.

### 1.3 Co-rumination and friendship quality

Co-rumination not only carries risks for internalizing problems but also confers benefits. It exhibits a positive association with friendship quality, wherein higher friendship quality allows adolescents to access greater social support, thereby mitigating internalizing symptoms (La Greca and Harrison, 2005). However, this relationship is complex. Longitudinal studies indicate a bidirectional and mutually causal linkage between the two (Felton et al., 2019; Rose et al., 2007), while other research identifies friendship quality as a moderator of the co-rumination–internalizing symptom association (Schwartz-Mette and Smith, 2018).

Evidence suggests gender and age may moderate the co-rumination–friendship quality dynamic. For instance, Rose (2002) reported a stronger link between co-rumination and positive friendship quality in males, and observed that this association emerges primarily in adolescents rather than children. However, findings remain inconsistent, with some studies failing to replicate gender- or age-related moderation effects (Felton et al., 2019). Given the complexity of the co-rumination–friendship quality relationship, the current study narrows its focus to co-rumination as the independent variable and friendship quality as the dependent variable, aiming to validate the beneficial pathway of co-rumination while testing the moderating roles of gender and age.

### 1.4 Co-rumination and Big Five personality

To date, no studies have explicitly investigated the relationship between co-rumination and personality trait types. This gap may be attributed to the field’s predominant focus on adolescent populations (Rose, 2021) and researchers’ general inclination to associate personality with more mature individuals (Soto and Tackett, 2015). However, evidence suggests that examining the interplay between adolescent interpersonal interactions and personality is both feasible and necessary, as adolescent personality and social interactions dynamically influence one another (Back et al., 2011; Soto and Tackett, 2015). Prior research also hints at potential links between personality characteristics and co-rumination. For instance, DiGiovanni et al. (2021) identified individual heterogeneity in the trade-off outcomes of co-rumination: some individuals are more likely to gain higher friendship quality without experiencing internalizing symptoms, while others are more prone to developing internalizing problems. Similarly, Tilton-Weaver and Rose (2023) utilized latent profile analysis to categorize co-rumination groups with varying degrees of benefits and risks. These findings suggest that personality traits may associate with distinct trade-off outcomes of co-rumination. Specifically, individuals scoring high on certain personality traits may be more susceptible to internalizing problems rather than achieving enhanced friendship quality through co-rumination. Clarifying the relationship between personality traits, co-rumination, and its associated outcomes, as well as identifying personality-based risk factors for co-rumination, would enable more effective identification and screening of high-risk co-rumination groups, thereby facilitating targeted interventions.

The Big Five personality traits are the most widely used and well-developed personality classification system, and they are also applicable to adolescent groups (Soto and Tackett, 2015). Therefore, this study uses the Big Five framework to preliminarily explore the relationship between co-rumination and personality in adolescents. Each of the five personality traits has a theoretical association with the specific structure of co-rumination: neuroticism is closely related to the internalizing symptoms brought about by co-rumination. Agreeableness and extraversion are the main factors influencing dyadic interactions with others (Cuperman and Ickes, 2009). Openness lacks a conceptual association with interpersonal interactions and is rarely used in studies of social interactions. However, co-rumination is a complex structure that is both individual and social in nature (Rose, 2002), and rumination, which conceptually overlaps with it, is considered related to an individual’s openness (Trapnell and Campbell, 1999). Therefore, it is necessary to explore the relationship between openness and co-rumination. Finally, individuals with high conscientiousness are believed to have stronger self-control abilities, which may lead to fewer internalizing problems after co-rumination (Jensen-Campbell and Malcolm, 2007).

At the same time, the Big Five change as individuals mature, and there are gender differences in various personality traits (Soto et al., 2011). As mentioned earlier, co-rumination behavior in individuals also shows developmental changes and gender differences: older adolescents are more likely to engage in co-rumination, and girls tend to co-ruminate more than boys. Why is this the case? Can the gender differences and age-related changes in co-rumination be explained by the differences in personality traits across gender and the maturation of these traits with age? This is a direction worth exploring.

### 1.5 Co-rumination in the context of Chinese culture

Research on co-rumination is primarily based on Western samples, and the results lack cross-cultural universality. Several scholars have called for examining co-rumination outcomes across diverse cultural contexts (Huang et al., 2022; Rose, 2021). In response to this call, the present study uses a sample of Chinese adolescents, who belong to a collectivistic culture, to rigorously validate previous findings.

### 1.6 Current study

**Figure 1:**
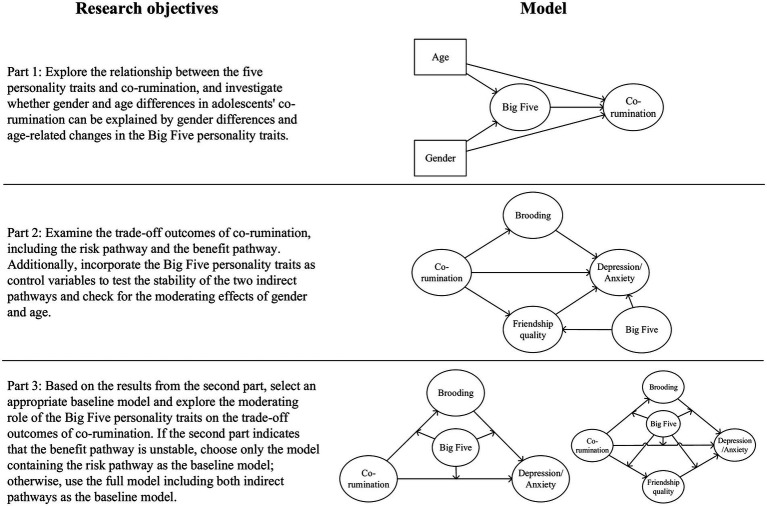
Research objectives of the three components and their corresponding models. For model simplicity, the “Big Five” in the diagram represents five personality traits, with each trait being a separate variable in actual modeling.

Based on the aforementioned discussion, this study not only validates previous research findings (including both confirmed and contradictory results) through more precise latent variable analysis using a Chinese adolescent sample, but also comprehensively explores the relationship between adolescents’ Big Five personality traits, co-rumination, and its consequential trade-offs through a three-part progressive analysis, while accounting for gender and age factors (see Figure 1). Firstly, the first objective of this study is to investigate the association between adolescents’ Big Five and co-rumination, attempting to explain gender and age differences in co-rumination through personality trait variations across gender and age groups. It aims to address two questions: Which personality traits are more/less likely to engage in co-rumination? Can gender and age differences in co-rumination be attributed to personality traits? Secondly, co-rumination exhibits trade-off effects. The second objective of this study is to explore whether co-rumination has unique associations with friendship quality and internalizing problems after controlling for the influence of Big Five personality traits, and to verify the moderating effects of gender and age groups. It attempts to answer the question: Are the trade-off outcomes resulting from co-rumination influenced by individual personality traits? Lastly, recent research has identified risk groups within co-rumination, where certain individuals are more likely to experience internalizing problems rather than high friendship quality (DiGiovanni et al., 2021; Tilton-Weaver and Rose, 2023). Therefore, the final objective of this study is to explore whether the Big Five can serve as indicators for identifying at-risk groups in co-rumination, attempting to answer the question: Which personality traits make individuals more or less likely to suffer from internalizing problems following co-rumination?

As an exploratory study, we do not make rigid hypotheses. However, drawing on previous findings, we can tentatively posit that: neuroticism, strongly linked to internalizing problems, may predict higher co-rumination and greater vulnerability to its adverse effects; agreeableness and extraversion, with their social attributes, may lead to more co-rumination; high openness may engage in more co-rumination driven by curiosity; conscientiousness, associated with self-regulation, may buffer against co-rumination-induced internalizing problems.

This study holds significant theoretical and practical implications in the field of co-rumination. Firstly, by utilizing a sample of Chinese adolescents characterized by collectivism, it further examines the cross-cultural generalizability of research findings primarily based on Western populations. Secondly, employing more precise latent variable analysis methods, this study is the first to test the measurement invariance of the co-rumination questionnaire, enabling us to understand the questionnaire’s effectiveness across different groups and guiding its future application in research. Thirdly, it comprehensively explores for the first time the relationship between the Big Five personality traits and co-rumination, helping researchers uncover potential connections between these traits and co-rumination while suggesting directions for deeper investigations. Lastly, this study seeks to identify personality indicators for recognizing at-risk groups for co-rumination, which can aid in screening such individuals in real-world settings to provide more targeted interventions.

## 2 Methods

### 2.1 Participants

The data for this study were collected from four schools in Nanning, Guangxi, China, with all participants being Chinese adolescents. Data from one of the schools were collected online using the questionnaire platform “Wenjuanxing,” while the remaining data were collected through students’ paper-and-pencil responses. The questionnaires were collected anonymously, with the first page of each questionnaire serving as an informed consent page. Data were only recorded after students read the informed consent page and explicitly wrote “I agree” at the end. To balance potential order effects during questionnaire completion, the presentation order of the scales was disarranged. Additionally, since adolescents exhibit more acquiescent responses when filling out questionnaires (Soto et al., 2008), three check items were included in the questionnaire, such as “Please select ‘Completely Disagree’ for this question.” Only data from participants who passed all check items were considered valid. A total of 1,018 samples were recorded, with 765 valid responses (75.1%). The female sample size was 384 (50.2%). The age range of the sample was 12 to 20 years, with one student aged 20 and six students aged 19; most students were 18 years old or younger, with an average age of 15.05 (*SD* = 2.16). The sample sizes for junior and senior high school students were 400 (52.3%, *M*_age_ = 13.138, SD = 0.791) and 365 (47.7%, *M*_age_ = 17.148, SD = 0.829) respectively.

### 2.2 Measures

The measurement tools used in this study have been proven by previous literature to be applicable to adolescent groups. However, due to the possibility of adolescents exhibiting noticeable acquiescent responses (Soto et al., 2008), some scales in the data may contain a few items that do not align with the theoretical structure or have low squared multiple correlation (SMC, i.e., the item reliability of each factor). Based on this, the study will first consider the fit indices of each scale, and if the fit indices are poor, items will be deleted according to theoretical considerations and item reliability.

In addition, this study employs item parceling for structural equation modeling (Little et al., 2002). Before parceling, it is necessary to determine the scale structure. So this study conducts second-order factor analysis for multidimensional scales. The acceptance criteria for second-order models reference the target coefficient proposed by Doll et al. (1994), where values closer to 1 indicate greater acceptability of the higher-order model. For model fit, this study used four indicators: comparative fit index (CFI), Tucker–Lewis index (TLI), root mean square error of approximation (RMSEA), and standardized root mean square residual (SRMR). Regarding reliability, we selects SEM-based reliability indicators, reporting construct reliability (CR) for unidimensional scales and *ω_h_* for multidimensional scales. These have several advantages over the traditional Cronbach’s *α* coefficient, such as not requiring the tau-equivalence assumption. These reliability evaluation standards are consistent with Cronbach’s *α*, where values greater than 0.7 are considered acceptable, and values greater than 0.8 indicate good scale reliability (Cheung et al., 2023).

Normality tests revealed non-normal distributions for some items. Consequently, confirmatory factor analyses (CFA) utilized the robust maximum likelihood estimator (MLR), reporting MLR-corrected *χ*^2^ values, and all chi-square difference tests were calculated using the corrected formula. After determining the scale items, an omnibus CFA incorporating all first-order factors was conducted to ensure items loaded appropriately on their respective scales. The final model demonstrated acceptable fit: *χ*^2^ (4679) = 8480.654, RMSEA = 0.033, CFI = 0.845, TLI = 0.836, SRMR = 0.051.

#### 2.2.1 Friendship quality

The original Friendship Quality Questionnaire was developed by Parker and Asher (1993). This study uses the Chinese short version of the Friendship Quality Questionnaire translated by Zhou et al. (2005). Participants were asked to evaluate their friendship quality of their best same-sex friend. The questionnaire consists of 18 items across six dimensions: companionship and recreation, validation and caring, intimate exchange, help and guidance, conflict resolution, and conflict and betrayal. The first five dimensions are categorized as positive friendship quality, while the last dimension is categorized as negative friendship quality (Rose, 2002). Previous research has shown that friendship qualities of different properties have different associations with co-rumination (Felton et al., 2019). To avoid increasing the complexity of the study, this study only uses the positive friendship quality items, totaling 15 items with a 5-point scale. One item was removed due to its high conceptual overlap with co-rumination and low SMC of only 0.175. The fit indices for the first-order five-factor positive friendship quality model are acceptable: *χ*^2^ (67) = 206.545, RMSEA = 0.052, CFI = 0.93, TLI = 0.905, SRMR = 0.038. The model including a higher-order positive friendship quality factor also showed acceptable fit: *χ*^2^ (72) = 218.098, RMSEA = 0.052, CFI = 0.927, TLI = 0.908, SRMR = 0.041, with a target coefficient of 0.947, indicating the second-order model is acceptable. The reliability *ω_h_* is 0.803. Hereafter, positive friendship quality will be referred to simply as friendship quality.

#### 2.2.2 Co-rumination

The co-rumination questionnaire was adapted from Rose (2002), and this study used the abbreviated version by Jose et al. (2012). The scale is considered unidimensional, consisting of 9 items with a 5-point scoring system. Participants were asked to evaluate their co-rumination scores when engaging in co-rumination with their best same-sex friend. The Chinese version referenced the translation by Yu et al. (2018), with minor modifications to some items to improve fluency while strictly maintaining the original meaning. One item was removed due to its poor fit and low SMC of 0.214. The unidimensional model demonstrated acceptable fit: *χ*^2^ (20) = 133.265, RMSEA = 0.086, CFI = 0.916, TLI = 0.882, SRMR = 0.046, with CR of 0.843.

#### 2.2.3 Brooding

This study employed the Brooding subscale of the Ruminative Response Scale (Treynor et al., 2003), originally developed by Nolen-Hoeksema and Morrow (1991) and translated by Han and Yang (2009). Brooding is considered a more negatively-oriented repetitive thinking style, closely related to internalizing. In contrast, the other subscale reflecting problem-solving and dealing with difficulties, reflection, is less associated with internalizing (Bastin et al., 2014). The subscale consists of 5 items with a 4-point scoring system. Results showed that the fit indices for the unidimensional model were acceptable: *χ*^2^ (5) = 61.517, RMSEA = 0.122, CFI = 0.935, TLI = 0.87, SRMR = 0.036, with a CR of 0.808.

#### 2.2.4 Depression

The Center for Epidemiologic Studies Depression Scale (CES-D) was developed by Radloff (1977). This study used the Chinese short version of CES-D translated by Zhang et al. (2010). This abbreviated version consists of 10 items scored on a 4-point scale, encompassing three dimensions: depressed affect, somatic symptoms, and positive affect. CFA showed good fit indices: *χ*^2^ (32) = 111.232, RMSEA = 0.057, CFI = 0.957, TLI = 0.904, SRMR = 0.036. As the scale only includes three dimensions, adding a second-order depression factor did not change the model’s degrees of freedom, resulting in fit indices identical to the first-order model. The *ω_h_* is 0.821.

#### 2.2.5 Anxiety

The measurement of adolescent anxiety uses the Self-Rating Anxiety Scale (SAD), developed by Zung (1971). The Chinese version employs the version provided by Dai et al. (2010), which has been shown to be well-suited for adolescent populations. The scale encompasses four dimensions: anxiety and panic, somatic control, vestibular sensations, and gastrointestinal/muscular sensations, with a total of 20 items (Olatunji et al., 2006). CFA showed acceptable fit indices: *χ*^2^ (164) = 412.804, RMSEA = 0.045, CFI = 0.923, TLI = 0.91, SRMR = 0.046. The second-order model also demonstrated a acceptable fit: *χ*^2^ (166) = 427.949, RMSEA = 0.045, CFI = 0.919, TLI = 0.907, SRMR = 0.048, with a target coefficient of 0.965. The scale’s reliability *ω_h_* is 0.763.

#### 2.2.6 Big Five

The measurement of the Big Five personality traits in Chinese adolescents was conducted using the Adolescent Five-Factor Personality Questionnaire developed by Zhou et al. (2000) and revised by Zou (2003). The questionnaire includes five dimensions: neuroticism, agreeableness, conscientiousness, extraversion, and openness, with a total of 50 items. it has been widely applied to Chinese adolescent populations (Zou, 2003). Based on fit indices, item reliability, and theoretical considerations, two items each were removed from extraversion, agreeableness, and conscientiousness, while one item was removed from openness. CFAs were performed separately for each personality trait, with fit indices all within acceptable ranges. Openness showed the poorest fit indices, with *χ*^2^ (20) = 101.87, RMSEA = 0.073, CFI = 0.926, TLI = 0.897, SRMR = 0.041; while neuroticism demonstrated the best fit, with *χ*^2^ (27) = 52.384, RMSEA = 0.035, CFI = 0.981, TLI = 0.975, SRMR = 0.026. The CR for each personality trait ranged from 0.792 to 0.864.

### 2.3 Analysis

This study used SPSS 26 for descriptive statistics and correlations, while Mplus 8.2 was employed for confirmatory factor analysis and structural equation modeling. Missing data accounted for less than 1% of the total and were handled using pairwise deletion in SPSS analyses. For Mplus modeling, full information maximum likelihood was used to estimate missing values. Additionally, due to the normality of item packages, maximum likelihood estimator was used for structural equation modeling. For multidimensional scales, internally consistent parcels were built, while balanced parcels were constructed for unidimensional scales (Little et al., 2002). Co-rumination was divided into 4 parcels, Brooding into 2 parcels, and each of the Big Five personality traits was divided into 3 parcels.

## 3 Results

### 3.1 Correlations and measurement invariance

The intercorrelations of all variables are shown in Table 1. Given co-rumination’s centrality to this study, latent mean comparisons were performed to rigorously assess gender and age differences. Following the steps provided by Putnick and Bornstein (2016), tests of configural, metric, and scalar invariance were conducted sequentially, followed by cross-group latent mean comparisons. During comparison, the latent mean of a reference group was fixed at 0, and the significance of the *z*-test was used to determine whether differences between groups were significant. The fit indices for gender and junior/senior high school measurement invariance are shown in Table 2. For model identification, latent means were fixed at 0 and variances at 1 when testing configural and metric invariance. Invariance was established when CFI differences between constrained and unconstrained models were <0.01.

**Table 1:** Intercorrelations of study variables.

Variable	1	2	3	4	5	6	7	8	9	10	11
1. Gender^a^											
2. Age	0.036										
3. Friendship quality	0.111^**^	−0.092^*^									
4. Co-rumination	0.081^*^	0.100^**^	0.431^**^								
5. Brooding	0.122^**^	−0.004	0.074^*^	0.276^**^							
6. Depression	0.110^**^	0.082^*^	−0.195^**^	0.119^**^	0.432^**^						
7. Anxiety	0.267^**^	−0.044	−0.073	0.188^**^	0.426^**^	0.745^**^					
8. E	−0.123^**^	−0.216^**^	0.349^**^	0.142^**^	−0.021	−0.302^**^	−0.184^**^				
9. A	0.049	−0.071	0.441^**^	0.178^**^	0.140^**^	−0.204^**^	−0.121^**^	0.452^**^			
10. C	−0.016	−0.141^**^	0.377^**^	0.110^**^	0.04	−0.313^**^	−0.221^**^	0.308^**^	0.557^**^		
11. O	−0.081^*^	−0.120^**^	0.374^**^	0.200^**^	0.161^**^	−0.159^**^	−0.025	0.459^**^	0.473^**^	0.518^**^	
12. N	0.194^**^	0.051	−0.028	0.212^**^	0.536^**^	0.597^**^	0.512^**^	−0.124^**^	0.039	−0.151^**^	−0.033

^*^p < 0.05, ^**^p < 0.01, two-tailed test. E = extraversion, A = agreeableness, C = conscientiousness, O = openness, N = neuroticism. The same applies below.

a Gender is a dummy variable, 1 = boys, 2 = girls. The same coding applies hereafter.

**Table 2:** Measurement invariance of co-rumination questionnaire.

Model	MLR*χ*^2^ (df)	RMSEA	CFI (ΔCFI)	TLI	SRMR	Δ*χ*^2^ (Δdf)	*p*	Decision
Gender								
Configural	151.164 (40)	0.085	0.917	0.884	0.049	—	—	—
Metric	163.361 (48)	0.079	0.914 (0.003)	0.900	0.055	6.716 (8)	0.568	Accept
Scalar	178.164 (54)	0.077	0.908 (0.006)	0.907	0.059	12.407 (7)	0.088	Accept
Age^a^								
Configural	149.971 (40)	0.085	0.922	0.89	0.048	—	—	—
Metric	160.796 (48)	0.078	0.920 (0.002)	0.906	0.057	5.100 (8)	0.747	Accept
Partial scalar	177.420 (54)	0.077	0.912 (0.008)	0.909	0.061	15.776 (6)	0.015	Accept
Scalar	185.255 (55)	0.079	0.907 (0.013)	0.906	0.062	24.697 (7)	<0.001	Reject

ΔCFI, Δ*χ*^2^ and Δdf are calculated by subtracting the corresponding values of the subsequent constrained model from those of the preceding freely estimated model. Both partial scalar invariance and scalar invariance models are compared with the metric invariance model.

a Ages were divided into junior and senior high school groups for comparison.

In cross-gender invariance testing, the co-rumination scale satisfied scalar invariance between boy and girl groups. For latent mean comparison, the boy group’s latent mean was fixed at 0, and the girl group’s latent mean was 0.181, SE = 0.08, *z* = 2.274, *p* = 0.023. Next, cross-group invariance measurement was conducted for junior and senior high school groups. Full scalar invariance was violated, partial scalar invariance was achieved by freeing one item intercept constraint. Latent mean comparison in the partial scalar invariance model showed marginally significant results. The junior high school group’s latent mean was constrained to 0, and the senior high school group’s latent mean was 0.156, SE = 0.081, *z* = 1.928, *p* = 0.054. Based on Ployhart and Oswald’s (2004) recommendation that if the focus is on latent mean differences and these differences theoretically exist, indicator intercepts can be constrained. Accordingly, all intercepts were constrained to be equal for latent mean comparison. The senior high school group’s point estimate was 0.192, SE = 0.081, *z* = 2.374, *p* = 0.018. The results indicated that the senior high school group had significantly higher levels of co-rumination.

### 3.2 The association between co-rumination and Big Five

**Figure 2:**
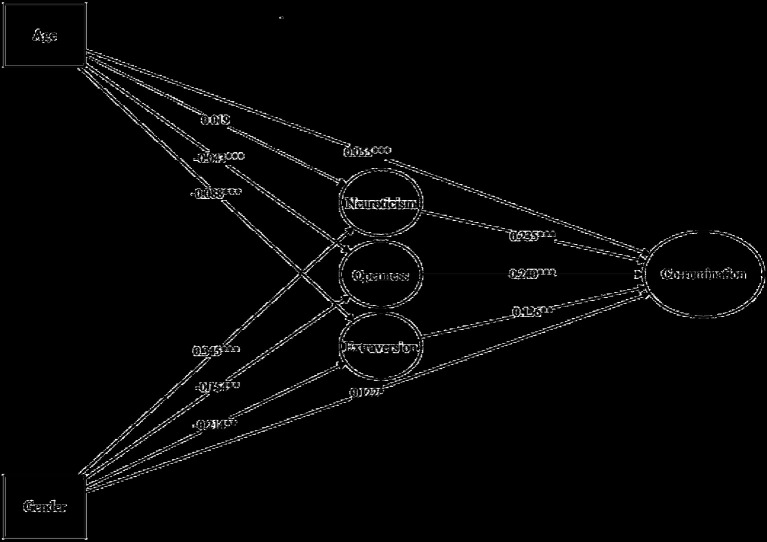
Mediation model with unstandardized path coefficients. ^*^p < 0.05, ^**^p < 0.01, ^***^p < 0.001, two-tailed test. For simplicity, only the structural model is presented, with the measurement model omitted. The correlation between gender and age was set to 0.

To address the two questions in the first research objective (i.e., which personality traits are more/less likely to engage in co-rumination? Can gender and age differences in co-rumination be attributed to personality traits?), this section constructed a mediation model with age and gender as initial independent variables, co-rumination as the final dependent variable, and the five personality traits as mediators. This study was exploratory, and the five personality traits were initially included in the model simultaneously. Personality traits unrelated to co-rumination (i.e., paths with non-significant “Big Five → co-rumination” coefficients) were removed, retaining the remaining personalities (final results shown in Figure 2). The model fit was acceptable, *χ*^2^ (81) = 449.085, RMSEA = 0.077, CFI = 0.922, TLI = 0.900, SRMR = 0.094. Results indicated that among the five personalities, neuroticism, extraversion, and openness positively predicted co-rumination. In the direct paths (i.e., “age/gender → co-rumination”), both path coefficients were significantly positive, reconfirming the age and gender differences in co-rumination and indicating that gender and age have unique associations with co-rumination after controlling for the three personality traits. Next, the entire indirect paths (i.e., “gender/age → N/O/E → co-rumination”) were examined. If both path coefficients were positive and significant, the personality trait was considered to explain the gender or age differences in co-rumination. Results showed that only the indirect path “gender → N → co-rumination” had both path coefficients positive and significant, indicating that gender differences in co-rumination can be partially attributed to gender differences in neuroticism. This study did not identify any personality traits that could explain the age-related developmental differences in co-rumination.

### 3.3 The relationship between the trade-off effect of co-rumination and Big Five

After examining the relationship between personality traits and co-rumination itself, the second part of the analysis will address the question: “Is the trade-off outcome resulting from co-rumination related to individual personality traits?” This study constructed a parallel mediation model with co-rumination as the exogenous variable, internalizing symptoms as the outcome variable, and friendship quality and brooding as mediators.

First, the trade-off model without control variables was tested. The model fit was good, with the model using depression as the outcome variable showing *χ*^2^ (72) = 238.203, RMSEA = 0.055, CFI = 0.960, TLI = 0.949, SRMR = 0.045, and the model using anxiety as the outcome variable showing *χ*^2^ (85) = 261.192, RMSEA = 0.052, CFI = 0.959, TLI = 0.950, SRMR = 0.043. The coefficients are presented in the M0 section of Table 3. The results confirm the trade-off effect of co-rumination in Chinese adolescents. The path coefficients for “co-rumination → brooding → anxiety/depression” were all positive and significant. The path coefficient for “co-rumination → friendship quality” was positive and significant, while the path coefficient for “friendship quality → depression/anxiety” was negative and significant.

**Table 3:** Unstandardized path estimates for parallel multiple mediator model.

Model	DV	IV	Depression			Anxiety		
			*B*	SE	*p*	*B*	SE	*p*
M0	Brooding							
		Co-rumination	0.285	0.039	<0.001	0.291	0.039	<0.001
	Friendship quality							
		Co-rumination	0.344	0.033	<0.001	0.343	0.033	<0.001
	Depression/Anxiety							
		Co-rumination	0.132	0.044	0.003	0.145	0.037	<0.001
		Friendship quality	−0.473	0.071	<0.001	−0.216	0.057	<0.001
		Brooding	0.541	0.045	<0.001	0.380	0.039	<0.001
M1	Brooding							
		Co-rumination	0.325	0.040	<0.001	0.327	0.040	<0.001
	Friendship quality							
		Co-rumination	0.300	0.031	<0.001	0.299	0.031	<0.001
		E	0.054	0.029	0.061	0.054	0.029	0.063
		A	0.216	0.054	<0.001	0.217	0.054	<0.001
		C	0.085	0.048	0.080	0.085	0.049	0.082
		O	0.038	0.042	0.370	0.038	0.042	0.369
		N	−0.078	0.025	0.002	−0.077	0.025	0.002
	Depression/Anxiety							
		Co-rumination	0.017	0.040	0.680	0.061	0.038	0.110
		Friendship quality	−0.140	0.075	0.061	−0.011	0.070	0.880
		Brooding	0.137^b^	0.042	0.001	0.138^b^	0.050	0.006
			0.308^g^	0.056	<0.001	0.353^g^	0.056	<0.001
		E	−0.122	0.036	0.001	−0.114	0.033	0.001
		A	−0.001	0.067	0.984	−0.019	0.063	0.759
		C	−0.205	0.059	0.001	−0.196	0.057	0.001
		O	0.088	0.051	0.088	0.206	0.049	<0.001
		N	0.375	0.037	<0.001	0.244	0.034	<0.001

b represents the path coefficient for the boys’ group in cross-group free estimation, while g represents the path coefficient for the girls’ group. DV, dependent variable; IV, independent variable.

To further clarify the relationship between co-rumination and its trade-off outcomes, the five personality traits were added as control variables to explain the variance in internalizing problems and friendship quality, forming Model M1. As mentioned earlier, numerous studies have already established the “co-rumination → brooding → anxiety/depression” pathway, so the “Big Five personality → brooding” pathway was not constructed to explain the remaining variance in brooding. The model fit was acceptable. For the model with depression as the outcome variable, the fit indices are *χ*^2^ (347) = 1251.755, RMSEA = 0.058, CFI = 0.915, TLI = 0.901, SRMR = 0.070. For the model with anxiety as the outcome variable, the fit indices are *χ*^2^ (375) = 1258.263, RMSEA = 0.055, CFI = 0.918, TLI = 0.905, SRMR = 0.068. The path coefficients are presented in the M1 section of Table 3. After controlling for internalizing variables, the “co-rumination → depression/anxiety” and “friendship quality → depression/anxiety” paths were no longer significant; the path coefficient for “brooding → depression/anxiety” remained significant. After controlling for friendship quality, the path coefficient for “co-rumination → friendship quality” remained significant. These results indicate that after adding the Big Five as control variables, the indirect pathway “co-rumination → brooding → depression/anxiety” remained robust with significant path coefficients, while the “co-rumination → friendship quality → depression/anxiety” indirect pathway was unstable, with the “friendship quality → depression/anxiety” pathway being non-significant. This may suggest that the risks of co-rumination outweigh its benefits.

To examine whether the trade-off effects of co-rumination differ by gender and age group, this study conducted multi-group comparisons for the five paths of the trade-off effect (i.e., “co-rumination → brooding,” “co-rumination → depression/anxiety,” “co-rumination → friendship quality,” “brooding → depression/anxiety” and “friendship quality → depression/anxiety”) based on Model M1. The model path coefficients were freely estimated across groups, with only one path constrained to be equal across groups at a time, followed by a chi-square difference test with 1 degree of freedom. A significant result indicates that the path coefficients differ across groups. First, in the gender cross-group estimation, significant differences were found in the path coefficients for “brooding → depression” Δ*χ*^2^ (1) = 9.222, *p* = 0.002, and “brooding → anxiety” Δ*χ*^2^ (1) = 6.892, *p* = 0.009. The point estimates and significance of these two path coefficients are shown in Table 3. Second, in the comparison between junior and senior high school groups, the chi-square difference test results indicated no significant differences in the five path coefficients between these groups.

### 3.4 Identifying at-risk groups for co-rumination through the Big Five

The third part of the study aims to answer the question, “What personality traits are more or less likely to be troubled by internalizing problems after co-rumination?” It explores the possibility of using Big Five as markers for identifying at-risk groups by constructing a moderated mediation model. The analysis in the second part has found that the risks of co-rumination outweigh the benefits, as it is more likely to lead to internalizing problems. The internal mechanism by which co-rumination causes internalizing issues was also clearly identified in previous literature. Therefore, this study selected the mediation path with a clearer causal relationship, “co-rumination → brooding → anxiety/depression,” and incorporated personality variables to build a moderated mediation model. Since this part is exploratory, the moderating variables were initially set to moderate the three paths of the mediation model, retaining the moderated mediation model with significant interaction term coefficients. The study used latent moderated structural equation (LMS) to construct interaction terms (i.e., using the XWITH code in Mplus). Due to the complexity and time consumption of calculations, not all five personality traits were simultaneously included as moderating variables in the model; instead, they were modeled separately. Following the guidelines provided by Maslowsky et al. (2015), after identifying the moderated mediation models, corresponding models without interaction terms were constructed to assess model fit indices and conduct log-likelihood ratio tests. The retained model results consistently showed good fit indices for the non-interaction models and significant log-likelihood ratio test outcomes. The path coefficients and moderating effects of each model are presented in Table 4. Results indicate that extraversion and neuroticism moderated the “brooding → depression/anxiety” path, with extraversion negatively moderating the relationship between brooding and internalizing, while neuroticism positively moderated this relationship. Additionally, conscientiousness and agreeableness played unique moderating roles, with adolescents’ conscientiousness negatively moderating the “brooding → depression” path, and agreeableness negatively moderating the “co-rumination → anxiety” path.

**Table 4:** Unstandardized path estimates for moderated mediation model.

Moderator
	DV	IV	Depression			Anxiety		
*B*			SE	*p*	*B*	SE	*p*
E	Brooding							
		Co-rumination	0.287	0.039	<0.001	0.291	0.039	<0.001
	Depression/Anxiety							
		Co-rumination	0.016	0.034	0.641	0.097	0.029	0.001
		Brooding	0.531	0.045	<0.001	0.376	0.038	<0.001
		E	−0.220	0.028	<0.001	−0.124	0.024	<0.001
		Brooding × E	−0.116	0.040	0.004	−0.083	0.033	0.013
N
	Brooding							
		Co-rumination	0.313	0.039	<0.001	0.313	0.039	<0.001
	Depression/Anxiety							
		Co-rumination	−0.085	0.032	0.007	0.030	0.028	0.287
		Brooding	0.273	0.044	<0.001	0.245	0.038	<0.001
		N	0.478	0.037	<0.001	0.306	0.033	<0.001
	Brooding × N	0.157	0.040	<0.001	0.151	0.038	<0.001
C
	Brooding							
		Co-rumination	0.288	0.038	<0.001	—	—	—
	Depression/Anxiety							
		Co-rumination	0.016	0.033	0.615	—	—	—
		Brooding	0.527	0.044	0.000	—	—	—
		C	−0.351	0.036	0.000	—	—	—
	Brooding × C	−0.120	0.051	0.019	—	—	—
A
	Brooding							
		Co-rumination	—	—	—	0.293	0.039	<0.001
	Depression/Anxiety							
		Co-rumination	—	—	—	0.097	0.029	0.001
		Brooding	—	—	—	0.399	0.039	<0.001
		A	—	—	—	−0.158	0.032	<0.001
	Co-rumination × A	—	—	—	−0.092	0.037	0.013

## 4 Discussion

### 4.1 The gender and age difference of co-rumination

Previous studies, primarily based on Western adolescent samples, have consistently shown gender and age differences in co-rumination among adolescents. This study, using a Chinese adolescent sample, employed more precise latent mean comparisons to verify these differences. Measurement invariance results indicate that the co-rumination scale can be effectively applied to both boy and girl groups, with gender differences in scale scores reflecting real differences in co-rumination. However, in cross-age measurement invariance tests, the junior and senior high school groups only achieved partial scalar invariance, meaning that true differences in co-rumination were only reflected in some items (Ployhart and Oswald, 2004). This may be due to differences in acquiescent responses and verbal comprehension abilities across age groups (Soto et al., 2008), resulting in different reference points for the two groups when answering certain items. Nevertheless, previous research has cross-validated through observational methods that higher age groups indeed engage in more co-rumination (Rose et al., 2022).

### 4.2 The relationship between Big Five and co-rumination, and their gender and age differences

This study is the first to explore the relationship between co-rumination in adolescents and the Big Five as well as the contribution of personality traits to developmental and gender differences in co-rumination. Results from the first structural equation model indicate that neuroticism, extraversion, and openness positively predict co-rumination. Possible reasons are as follows. Firstly, neuroticism is associated with an individual’s negative bias and attachment anxiety. On one hand, individuals high in neuroticism are more sensitive to negative information in their environment and more likely to detect it, providing more topics for co-rumination (Pang and Wu, 2023). On the other hand, they are more prone to developing attachment anxiety (Crawford et al., 2007), which may drive them to engage in more co-rumination with peers. Simultaneously, their poorer cognitive control abilities make it difficult for them to disengage from these negative topics (Munoz et al., 2013).

Secondly, extraversion is closely related to social attributes, with highly extraverted individuals having a broader social network (Selfhout et al., 2010). They may actively participate in various forms of social activities, including co-rumination, due to their social needs. Additionally, their higher friendship quality with peers also promotes the occurrence of co-rumination, which involves self-disclosure (Felton et al., 2019; Grossmann et al., 2023).

Finally, individuals high in openness are more receptive to new experiences and may engage in co-rumination activities due to their strong curiosity. The findings of this study may suggest that even curiosity-driven behaviors that are neutral in nature can lead to negative effects (i.e., co-rumination). Of course, this requires further exploration of different types of co-rumination. Trapnell and Campbell (1999), in their study of personality traits and repetitive thinking, distinguished between neutral repetitive thinking (reflection) driven by openness and negative repetitive thinking (rumination) driven by neuroticism, with rumination being pathological and leading to more negative psychological effects compared to reflection. Bastin et al. (2014) also differentiated between different types of co-rumination: neutral co-rumination (co-brooding) and negative co-rumination (co-reflection). Future researchers need to further verify whether individuals high in openness are more likely to engage in neutral co-rumination rather than negative co-rumination.

In addition, this study also attempted to explore whether gender and age differences in co-rumination are related to personality traits. Regarding gender differences, analysis revealed that neuroticism could partially explain why co-rumination occurs more frequently in girls. Girls tend to have higher levels of neuroticism, which suggests they are likely to experience stronger attachment anxiety or pay more attention to negative information, thus increasing the possibility of co-rumination. In terms of age, the study could not identify individual trait-level variables that explain the developmental changes in co-rumination. This may indicate that age differences in co-rumination are more likely due to adolescents’ social development and environmental factors rather than personal personality factors. For example, from the perspective of adolescents’ social development, as adolescents mature physically and mentally, they increasingly desire to break away from their families and explore the world, prompting them to interact more spontaneously with peers and gradually reduce communication with parents (Rose and Rudolph, 2006). Considering the social environmental factors in China, senior high schools more often adopt a boarding school format compared to junior high schools, providing adolescents with more opportunities for peer interaction.

Overall, adolescents with higher levels of neuroticism, openness, and extraversion demonstrate higher levels of co-rumination. Gender differences in co-rumination can be primarily explained by gender differences in neuroticism, with girls typically scoring higher in neuroticism than boys, making them more prone to co-rumination (i.e., “gender → N → co-rumination”). However, age-related changes in co-rumination are more dependent on social factors rather than individual traits.

### 4.3 Big Five personality and the trade-off effect of co-rumination

After clarifying the personality factors that may affect individual co-rumination, this study explored the trade-off outcomes of co-rumination. In addition to verifying the pathways of trade-off outcomes, it also clarified the influence of personality traits on this trade-off effect. The research results successfully revealed the trade-off pathway: adolescents increased their post-event brooding through co-rumination behavior, thereby increasing the risk of internalizing symptoms; simultaneously, co-rumination improved the friendship quality with their best same-sex friend, and this closer friendship provided individuals with more social support while protecting against the risk of internalizing problems.

To further explore this relationship, the present study incorporated the Big Five personality traits as control variables into the model. The results showed that, after controlling for the relationship between the Big Five and friendship quality, co-rumination still positively predicted friendship quality. This indicates that the relationship between co-rumination and friendship quality is robust and unique, and that co-rumination behavior involving self-disclosure can effectively enhance peer friendships. Additionally, the model also controlled for personality traits in relation to internalizing problems. Friendship quality no longer showed a protective effect on internalizing symptoms, suggesting that the prevention of internalizing problems depends more on individual personality factors. Brooding still significantly predicted an increase in internalizing problems, indicating that the relationship between post-event brooding triggered by co-rumination and internalizing problems is clear and stable. Overall, compared to the beneficial pathway of co-rumination (“co-rumination → friendship quality → internalizing problems”), the risk pathway of co-rumination (“co-rumination → brooding → internalizing problems”) appears more stable. It is not difficult to see that the risks of co-rumination outweigh its benefits, meaning that co-rumination is more likely to lead to an increase in internalizing problems after controlling personal trait factors.

Furthermore, this study compared the trade-off results of co-rumination between boys and girls, as well as between junior and senior high school students. The results indicated that girls are more likely to suffer emotional distress due to brooding. In conjunction with previous discussion, the study found that girls have a susceptibility to internalizing symptoms at various stages: they exhibit higher neuroticism, coupled with a social inclination for small-scale interactions unique to girls, leading to more co-rumination. Subsequently, the increase in co-rumination results in more individual brooding, and girls are also more prone to internalizing problems due to brooding. Based on this, the study clearly demonstrates the internal mechanism by which adolescent females are more likely to develop internalizing symptoms due to co-rumination.

In summary, the second part of the study validated the trade-off effect of co-rumination among Chinese adolescents, but the risks associated with co-rumination were more apparent. The high friendship quality resulting from co-rumination could not effectively prevent adolescents’ internalizing problems, and the mitigating effect of friendship quality on internalizing symptoms was more attributed to personality factors. Conversely, the brooding caused by co-rumination could independently predict adolescent internalizing problems, regardless of personality factors. Lastly, girls were identified as a risk group for co-rumination, with stronger associations between co-rumination-induced brooding and internalizing symptoms.

### 4.4 The risk and protective factors in co-rumination

After analyzing the second part, it was found that the risks associated with co-rumination are more apparent, and the internal mechanism leading to these risks is relatively clear (i.e., “co-rumination → brooding → internalizing problems”). Based on this, the final part of this study explores the moderating effect of the Big Five personality traits on this pathway, investigating the possibility of using the Big Five personality framework to identify group at risk for co-rumination.

Research findings suggest that high neuroticism is a risk factor for co-rumination: individuals with high neuroticism characterized by low cognitive control are more susceptible to internalizing symptoms due to brooding. Previous sections have also indicated that individuals with high neuroticism are often girls, which corresponds to the results in the second part, namely that girls are more likely to develop internalizing problems due to brooding, possibly because they tend to have higher levels of neuroticism.

Secondly, extraversion may be a protective factor against internalizing problems in adolescents. Highly extraverted individuals tend to gain energy and psychological support from the external world and maintain a more optimistic attitude toward it (Calvete et al., 2016). Simultaneously, they possess greater cognitive flexibility, allowing them to view and handle situations from more diverse perspectives, thus weakening the risk of internalizing associated with brooding (Chen et al., 2022).

Additionally, conscientiousness buffers the connection between brooding and depression, possibly related to the higher self-control and attention regulation abilities of highly conscientious individuals (Jensen-Campbell and Malcolm, 2007). These abilities regulate the attentional bias toward negative self-information caused by brooding, thereby reducing the risk of depression. However, for anxiety, anxious adolescents often worry excessively about self-related issues (Weems et al., 2000), and highly conscientious individuals who are highly responsible for themselves do not reject such worries, which may explain why the moderating effect of conscientiousness exists only for depression but not anxiety.

Finally, unexpectedly, this study also discovered the unique moderating effect of agreeableness. Individuals with high agreeableness were able to effectively buffer anxiety problems in adolescents following co-rumination. A possible explanation for this is that highly agreeable individuals tend to have higher trait mindfulness, experiencing less perceived stress during co-rumination (Duggan et al., 2024), which in turn reduces the likelihood of conditional anxiety induced by co-rumination (Dirghangi et al., 2015).

### 4.5 Summary

Based on the aforementioned results and discussion, several conclusions can be drawn. Firstly, the risks of co-rumination outweigh the benefits; compared to the protective effect of friendship quality, the brooding triggered by co-rumination more consistently increases internalizing risks. Secondly, neuroticism serves as an effective target for identifying at-risk groups in co-rumination. Gender differences in neuroticism effectively explain variations in co-rumination levels and the association between co-rumination and internalizing symptoms. Individuals with higher neuroticism are more prone to co-rumination and more susceptible to its negative effects. Thirdly, conscientiousness and agreeableness act as protective factors in co-rumination. Individuals with high conscientiousness and agreeableness are less likely to develop depression/anxiety problems during co-rumination. Additionally, the relationship between extraversion and co-rumination is more complex: on one hand, highly extraverted individuals actively engage in co-rumination with peers due to social needs; on the other hand, their more optimistic mindset and stronger cognitive flexibility make them less vulnerable to the negative consequences of co-rumination. Lastly, individuals with high openness, driven by curiosity, engage in more co-rumination. However, whether co-rumination behavior motivated by self-exploration is pathological and leads to negative outcomes requires more detailed future research.

### 4.6 Practical implications

First, this study found that adolescents of different age groups may have linguistic comprehension differences and varying acquiescence responses when completing co-rumination questionnaires, which may account for some of the score differences. This reminds researchers, especially those focusing on developmental changes in co-rumination, to ensure the comprehensibility and fluency of language when designing co-rumination questionnaires, while also including check items to reduce acquiescence responses. Additionally, it is advisable to employ trained administrators during assessments to provide clarifications on item meanings. Second, this study examined the relationship between the Big Five and co-rumination. We suggest using several indicators from the Big Five to jointly assess the risk level of individuals prone to co-rumination. Specifically, individuals at higher risk for depression could be screened using high neuroticism and low conscientiousness, whereas those at higher risk for anxiety could be identified through high neuroticism and low agreeableness. Of course, it is important to acknowledge the limitations inherent in this cross-sectional study. Therefore, we urge researchers to employ diverse methodologies to further validate and enrich these findings.

### 4.7 Limitations

Although this study carries substantial practical significance, it also exhibits notable limitations. Firstly, the research employs a cross-sectional design, meaning the relationships between variables only reflect a static time point and fail to capture the dynamic interplay between co-rumination and other variables. DiGiovanni et al. (2023) suggest that co-rumination is better understood as a state variable, so future studies should adopt designs that track temporal changes, such as cross-lagged or ecological momentary designs, to explore the dynamic relationship between personality and co-rumination. Secondly, the reliance on self-reported data introduces potential subjective biases. Future research could incorporate newly developed experimental paradigms to explore the association between co-rumination and personality (Tudder et al., 2023). Thirdly, this study did not conduct dyadic matching analyses between peers, missing the opportunity to examine co-rumination and personality traits from an interpersonal interaction perspective. Future work could employ more dyadic designs and analytical methods, such as the actor-partner interdependence model or the social relations model (Kenny et al., 2006), to better understand the role of personality in co-rumination as a social interactive behavior. Lastly, the findings regarding the relationship between the Big Five personality traits and co-rumination, as well as the measurement invariance of the co-rumination questionnaire, may be specific to Chinese adolescent samples and lack generalizability. Thus, Western samples should be used to further validate these results.

## Data Availability

The raw data supporting the conclusions of this article will be made available by the authors, without undue reservation.
